# Plant proteases: from molecular mechanisms to functions in development and immunity

**DOI:** 10.1093/jxb/erab129

**Published:** 2021-04-13

**Authors:** Renier A L van der Hoorn, Marina Klemenčič

**Affiliations:** 1 Plant Chemetics Laboratory, Department of Plant Sciences, University of Oxford, Oxford, UK; 2 Department of Chemistry and Biochemistry, Faculty of Chemistry and Chemical Technology, University of Ljubljana, Ljubljana, Slovenia

**Keywords:** Development, immunity, protease, regulation, substrate

**Plant proteases are important but enigmatic players in almost all aspects of plant life. Plant genomes encode hundreds of proteases that act in development, homeostasis, biotic and abiotic stress, symbiosis, and growth. Even though they act in all subcellular compartments and in all plant organs, their regulation and downstream actions are still poorly understood. Activities of proteases are tightly regulated because their action on proteins is irreversible. Moreover, as most proteases cleave multiple substrates, their modes of action are challenging to predict. The reviews and original papers in this issue highlight the latest findings in the field of plant protease research related to these enzymes in organisms ranging from Chlorophyta to angiosperms.**

Proteins represent the largest group of macromolecules in any living cell, including a plant cell. Precise regulation of their turnover is therefore of utmost priority for the cell. It is enzymes that use proteins as substrates—proteases that play the most important role in this process. They are the key players in maintaining cell homeostasis, and one of the processes that enables this is autophagy, where dysfunctional cellular components including proteins and organelles are recycled (Bozhkov, 2018). A key step in the formation of autophagosomes is lipidation of the ubiquitin-like protein ATG8, a reversible process, which is mediated by papain-like cysteine protease ATG4. Pérez-Pérez and colleagues describe how protease ATG4 (family C54 of clan CA) integrates redox and stress signals to regulate autophagy. ATG4 does so by initially activating the ATG8 protein by cleavage as well as recycling it by processing the amide bond between a cysteine residue on ATG8 and phosphatidylethanolamine. While the redox state of ATG4 is proposed to be the main regulator of its proteolytic activity, the role of other post-translational modifications, including phosphorylation, *S*-persulfidation, and *S*-nitrosylation, is critically reviewed (Pérez-Pérez *et al.*, 2021). Phosphorylation, as a mean of post-translational regulation in addition to ubiquitination, is controlling the function of another protease—the growth-restricting protease DA1. Chen *et al.* (2021) review the molecular mechanisms that fine-tune the activity of DA1 and therefore final organ size. Arabidopsis DA1 null mutants (*da1-1*) exhibit larger organs, including flowers, siliques, seeds, and leaves, which makes the DA1 protease one of the promising targets to increase crop yields in biotechnology and agriculture.

## Proteases enhance plant responses to biotic and abiotic stress

In addition to genetically encoded factors, which govern the size of plants and the biomass produced, crop productivity is strongly affected by external predators that plants have to fight against in order to survive. One of the most important crops is the grapevine, *Vitis vinifera* L., whose fruits are used for production of wine. The International Organisation of Vine and Wine estimated that the total amount produced in 2020 was 258 million hectolitres. Pathogen-mediated losses in wine production are substantial and are caused by oomycetes, fungi, and bacteria. The review by Santos and Figueiredo (2021) discusses two sides of the same story in grapevine–pathogen interactions, by considering the proteases that both the host and pathogens employ during infections. They describe the diverse proteases that grapevine plants accumulate during infection and their possible role, and summarize current knowledge of proteases used by grapevine pathogens during infection (Santos and Figueiredo, 2021).

The apoplast, the extracellular matrix of the plant, is the first territory that plants have to defend from invading pathogens. It therefore contains a plethora of proteases, potentially involved in immunity. Godson and van der Hoorn (2021) report a meta-analysis of 46 apoplastic proteases involved in plant immunity and critically evaluate them against criteria for apoplastic immune proteases, highlighting gaps in our knowledge and the need for more careful definitions. This review also classifies six mechanisms through which these apoplastic immune proteases tend to act.

However, pathogens also use their proteolytic arsenal, which is aimed at damaging the intracellular proteostasis of the host. In parallel to plant proteases, this field of knowledge has also steadily grown. The review by Mooney *et al.* (2021) summarizes the manipulation of plant immunity by bacterial Type III effector proteases. Although these are not plant proteases, their regulation and substrates are inside plant cells. This review describes 19 proteases that bacterial plant pathogens inject into the host cell, often to interfere with host immune signalling. Substrates for some of these effectors are known, and this knowledge is compared with that of mammalian bacterial pathogens, highlighting the need for unbiased approaches towards substrate identification of these protease effectors.

**Fig. 1.**
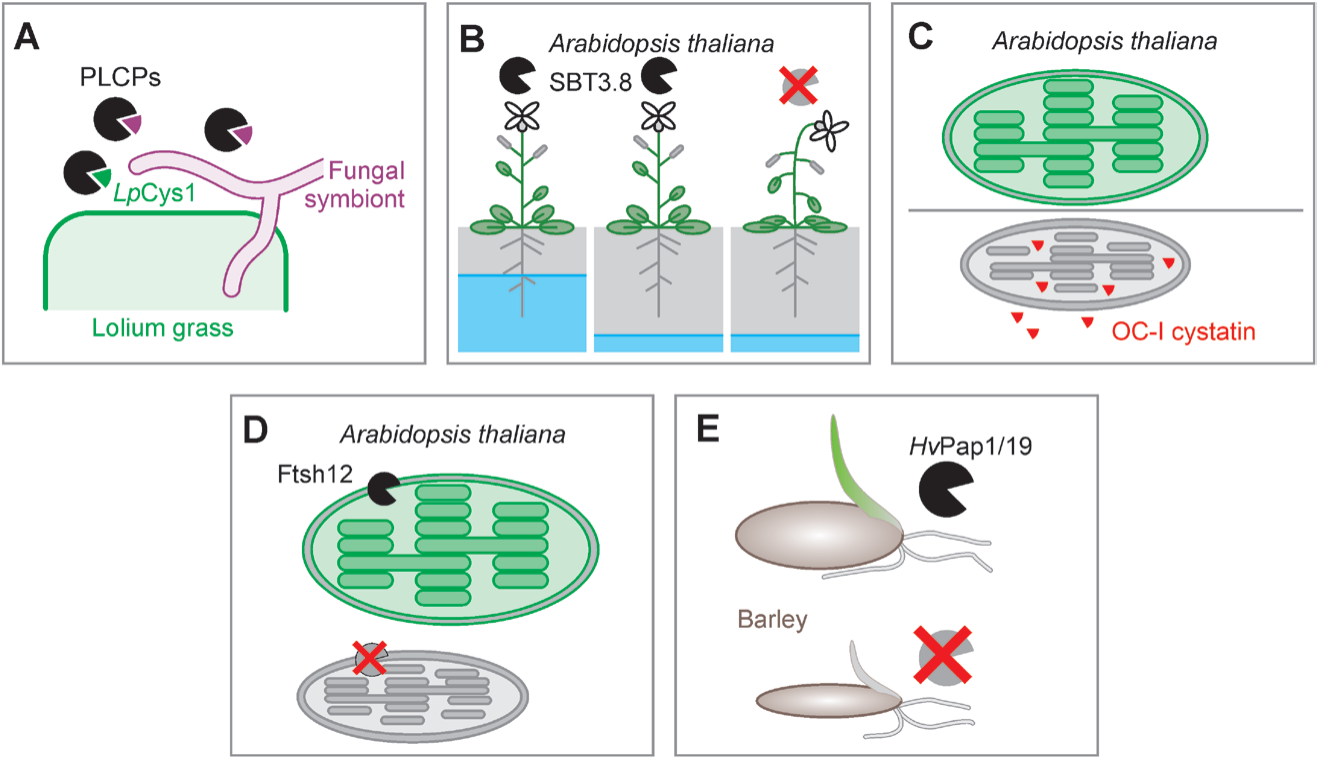
Diversification of the roles of proteases in plants. (A) Apoplastic papain-like cysteine proteases (PLCPs) of *Lolium* grass are suppressed upon colonization by the mutualistic fungal symbiont *Epichloë festucae*. Suppression of one PLCP might be caused by the plant cystatin LpCys1. (B) A*rabidopsis thaliana* lacking subtilase SBT3.8 is less drought tolerant. SBT3.8 is processing the phytosulfokine precursor. (C) *Arabidopsis thaliana* expressing cytoplasmic or chloroplastic cystatin OC-I have reduced chloroplast proteins and are less tolerant to high light stress. (D) *Arabidopsis thaliana* lacking the chloroplast-localized protease Ftsh12 have deformed chloroplasts, implicating a role for this protein in regulating chloroplastic proteins. (E) Depletion of two papain-like cysteine proteases, HvPap1 and HvPap19, from barley results in smaller seeds and delayed seed germination.

In addition to pathogens, mutualistic microorganisms also interfere with plant defences to maintain endophytic colonization with their hosts. Passarge and colleagues investigate the suppression of secreted plant papain-like cysteine proteases (PLCPs) of grasses upon infection with a symbiotic fungus. By performing activity-based protein profiling, they identified the active apoplastic papain-like proteases and investigated a plant cystatin, a proteinaceous inhibitor of papain-like cysteine proteases, that might be responsible for the suppression of some of these proteases (Fig. 1A). The suppression of secreted proteases is similar to observations made for various plant–pathogen interactions, suggesting that mutualistic and pathogenic microbes use similar strategies to avoid proteolysis when colonizing the host (Passarge *et al.*, 2021).

In order to survive and reproduce, plants, in addition to biotic invaders, also have to endure and overcome abiotic stress conditions. Stührwohldt *et al.* (2021) explore the role of a serine protease subtilase SBT3.8 for production of a peptide hormone, which contributes to stress tolerance. They show that during stress, the precursor of the phytosulfokine peptide hormone is processed by SBT3.8 to enhance drought resistance in Arabidopsis. SBT3.8 is responsible for the C-terminal release of the PSK1 pentapeptide from its precursor by cleaving before an aspartate residue. This mechanism explains why *sbt3.8* mutants have a drought stress phenotype (Fig. 1B) that can be compensated by addition of recombinantly expressed and purified PSK1 protein.

## Development of organelles, organs, and organisms depends on proteolytic events

The broad role of PLCPs and the importance of the interaction with their inhibitors, cystatins, in the intracellular context is further demonstrated by Alomrani *et al.* (2021). By using transgenic Arabidopsis lines that accumulate a rice cystatin either in the cytoplasm or in the chloroplast they showed that papain-like cysteine proteases are involved in the regulation of the photosynthetic machinery. Both of these lines flowered later and had reduced protease activity, associated with higher levels of photosynthetic proteins, indicating a role for cystatin-targeted proteases in the degradation of chloroplast proteins (Fig. 1C). Also crucial for plastid development and thus proper function of the chloroplast is an ATP-dependent metalloprotease FtsH12, as described by Mielke *et al.* (2020). These authors suggest that FtsH12 participates in protein translocation into the chloroplast. Mutant *ftsh12* plants have small, deformed chloroplasts, and proteomic analyses confirmed the impairment in plastid development (Fig. 1D). Remarkably, however, N-terminome analysis did not display altered processing of plastid-imported proteins in the absence of FtsH12, despite the role of FtsH12 as a protease.

Moreover, PLCPs seem also to be crucial for seed germination. A study by Gomez-Sanchez *et al.* (2021) describes that the depletion of two PLCPs from barley by RNAi alters grain composition as they contain fewer carbohydrates but more proteins. Furthermore, these RNAi lines produce smaller grains that germinate later (Fig. 1E). Interestingly, the authors also noticed an increased chymotrypsin-like activity, attributed to the activity of serine proteases, hinting at possible compensatory mechanisms through interfamily protease redundancy.

## Future directions

We are witnessing dramatic progress in the field of plant proteases over recent years, demonstrating their role in symbiosis, chloroplast biogenesis, growth, drought stress, and defence in various photosynthetic organisms ranging from unicellular algae to flowering plants. Elucidating the molecular mechanisms of these enzymes is, however, an interdisciplinary task that requires collaboration of research teams with different expertise. Conferences where plant protease researchers can meet and initiate these collaborations are of great importance for the progress of this exciting field of research. The European plant protease community last met in Ghent in 2018 and the next meeting was originally planned for September 2020 in Ljubljana, Slovenia, but has been postponed to autumn 2022 due to the COVID-19 pandemic. Witnessed by the topics of the articles published in this special issue, we expect the field to tackle two of the main challenges of the protease research field in the future: its regulation and its substrates.
